# Extracting spatial-temporal features that describe a team match demands when considering the effects of the quality of opposition in elite football

**DOI:** 10.1371/journal.pone.0221368

**Published:** 2019-08-22

**Authors:** Bruno Gonçalves, Diogo Coutinho, Juliana Exel, Bruno Travassos, Carlos Lago, Jaime Sampaio

**Affiliations:** 1 Research Center in Sports Sciences, Health Sciences and Human Development (CIDESD), CreativeLab Research Community, Vila Real, Portugal; 2 Universidade de Trás-os-Montes e Alto Douro, Vila Real, Portugal; 3 Universidade da Beira Interior, Covilhã, Portugal; 4 Faculty of Education and Sport, University of Vigo, Pontevedra, Spain; University of Brasilia, BRAZIL

## Abstract

Spatiotemporal patterns of play can be extracted from competitive environments to design representative training tasks and underlying processes that sustain performance outcomes. To support this statement, the aims of this study were: (i) describe the collective behavioural patterns that relies upon the use of player positioning in interaction with teammates, opponents and ball positioning; (ii) and define the underlying structure among the variables through application of a factorial analysis. The sample comprised a total of 1,413 ball possession sequences, obtained from twelve elite football matches from one team (the team ended the season in the top-5 position). The dynamic position of the players (from both competing teams), as well as the ball, were captured and transformed to two-dimensional coordinates. Data included the ball possession sequences from six matches played against top opponents (TOP, the three teams classified in the first 3 places at the end of the season) and six matches against bottom opponents (BOTTOM, the three teams classified in the last 3 at the end of the season). The variables calculated for each ball possession were the following: ball position; team space in possession; game space (comprising the outfield players of both teams); position and space at the end of ball possession. Statistical comparisons were carried with magnitude-based decisions and null-hypothesis analysis and factor analysis to define the underlying structure among variables according to the considered contexts. Results showed that playing against TOP opponents, there was ~38 meters game length per ~43 meters game width with 12% of coefficient of variation (%). Ball possessions lasted for ~28 seconds and tended to end at ~83m of pitch length. Against BOTTOM opponents, a decrease in the game length with an increase in game width and in the deepest location was observed in comparison with playing against TOP opponents. The duration of ball possession increased considerable (~37 seconds), and the ball speed entropy was higher, suggesting lower levels of regularity in comparison with TOP opponents. The BOTTOM teams revealed a small EPS. The Principal Component Analysis showed a strong association of the ball speed, entropy of the ball speed and the coefficient of variation (%) of the ball speed. The EPS of the team in possession was well correlated with the game space, especially the game width facing TOP opponents. Against BOTTOM opponents, there was a strong association of ball possession duration, game width, distance covered by the ball, and length/width ratio of the ball movement. The overall approach carried out in this study may serve as the starting point to elaborate normative models of positioning behaviours measures to support the coaches’ operating decisions.

## Introduction

One of the biggest challenges in team sports is to validate performance indicators that contribute to optimize the coaching process and competition outcome [1]. Within the traditional methods of performance analysis in team sports, the notational analysis has been used to obtain indicators of discrete actions and/or events by using advanced statistical procedures [2, 3]. This approach allows to understand the static complexity of performance, to produce a valid and reliable description of individual and team behaviours and to describe teams’ performance by correlating a wide range of variables [4, 5]. Also, the players’ physical and physiological competitive demands [6] and their comparison with training have been incessantly investigated over the last years, allowing to identify different match profiles according to the distances covered at different speed thresholds [7–9]. The identification of such profiles brought relevant aspects to plan the physical loads from short to mid-term planning guidelines and also to minimize the fatigue and the risk of injuries [10, 11].

However, as such variables and methods describe the match results, they are limited in underpinning behaviours that lead to understand or even predict the outcomes. In line with these concerns, the players’ position data has recently emerged as one of the key determinants of team sports performance [12–15]. Such analysis allows to capture the players’ and teams’ spatiotemporal dynamics at different levels of analysis (from individuals to teams) with the integration of the contextual circumstances. Accordingly, several new instruments, procedures, processing techniques, and new visuals may be incorporated into the performance analysis scope to complement the static complexity analysis and attend the new dimension of questions related to the dynamic complexity.

In fact, the training process in modern association football is a multifactorial process that requires high complementary among physiological, technical and tactical workload prescriptions. To accomplish these goals, contextual information, such as field location, team strategy and opponent behaviour, that sustain players and teams’ tactical behaviour should be integrated in the analysis of physical loads [16–18]. That is, players’ physical demands over competition are constrained by their positioning dynamics [1, 19] that sustain the individual and collective tactical behaviour [20]. Tactical behaviour of players and teams are regulated by spatial-temporal informational constraints within the teams’ collaborative principles, that consequently, affect match physical demands. In this line of reasoning, previous ideas and methods from dynamical systems have been used to integrate different dimensions of analysis [21–24] and to explain how system working parts are connected and how they continuously adapt over time [1, 25]. The main idea is that the players and teams’ behaviours should be considered as a whole, where systems with many dynamically interacting elements are capable of wide-ranging patterns of behaviour [22]. In this regard, substantial advances have been made to increase the processing techniques aiming to assess regularities in players and teams’ positioning derived data [26, 27]. The entropy computations serve this purpose by allowing to measure the probability that the configuration of one segment of data in a time series will allow to predict the configuration of another segment of the time series a certain distance apart [28]. This technique has been used to identify if players’ positioning dynamics express a predictable pattern which may provide insights about the local information sources that are being used in their match decisions [26, 29–31]. Also, recent research has developed metrics to identify the determinants of collective behaviour that may optimise the players’ ability to attune their decisions within teammates during mutual tasks [12, 15]. For instance, the entropy of players’ distance to the centroid was suggested to be an order parameter that represents the use of local information in the decision making and team-related behaviours [26, 29]. Other metrics from the match-level of analysis, such as teams’ length and width, have been used to provide information regarding space occupation [32, 33].

The identification of individual and collective patterns of play according to game environment and teams’ collaborative principles over competition allow coaches to improve the process of planning, designing and executing training tasks. In fact, match analysis allows to extract relevant and transferable information from the competition to practice. Based on the information derived from competition, the training tasks should ensure a representative design, i.e., training is intended to represent the competitive context so practitioners can experience the similar perceptual-motor relations landscape, and exploit the inherently adaptive nature of their perceptual systems in their interactions with environments [34–36]. The main goal is to improve the transfer between technical, physical and individual and collective tactical behaviours from training to competition by sampling the informational constraints that players use to perform under the competitive environment [37]. Therefore, variables such as space of play, time, numerical relations, distance to the goal or even ball trajectory might play an important role in assisting the fundamentals of coaching.

Therefore, the aim of this study was to extract spatial-temporal patterns of play that characterize a team match demands when considering the effects of the quality of opposition. To fulfill this main goal, the approach was carried on a twofold step: (i) describe the collective behavioural patterns that relies upon players’ positioning in interaction with teammates, opponents and ball position; (ii) and define the underlying structure of the derived variables through application of a factorial analysis. It was hypothesized that accounting for the quality of opponents might reveal important variations in team behaviour as a function of ball possession. This information is key for tactical planning and would enhance the transferability of match demands to training tasks. Also, this approach may serve as the basis to elaborate normative models of positioning behaviour measures to support coaches’ decisions.

## Materials and methods

### Sample and data collection

The sample of this study comprised a total of 1,413 ball possession sequences, obtained from twelve elite football matches from English Premier League. The dynamic position of the players (from both competing teams), as well as the ball, was captured and transformed to two-dimensional coordinates using the TRACAB Optical Image Tracking System at 25 Hz. The system uses super-HD cameras and patented image processing technology to deliver live tracking of all moving objects with a maximum delay of just three frames (https://chyronhego.com/) and its used continuously in several leagues (English Premier League, German Bundesliga and Spanish La Liga) and also provided data for several studies [38, 39]. Data included the ball possession sequences from the six matches of one team when playing against top opponents (TOP, the three top teams classified in the top-3 at the end of the season) and six matches against bottom opponents (BOTTOM, the three bottom teams classified in the bottom-3 at the end of the season). The team ended the season in the top-5 position. The study protocol was approved and followed the guidelines stated by the Ethics Committee of the of University of Trás-os-Montes and Alto Douro, based at Vila Real (Portugal) and conformed to the recommendations of the Declaration of Helsinki.

### Processing and variables

**Fig 1:**
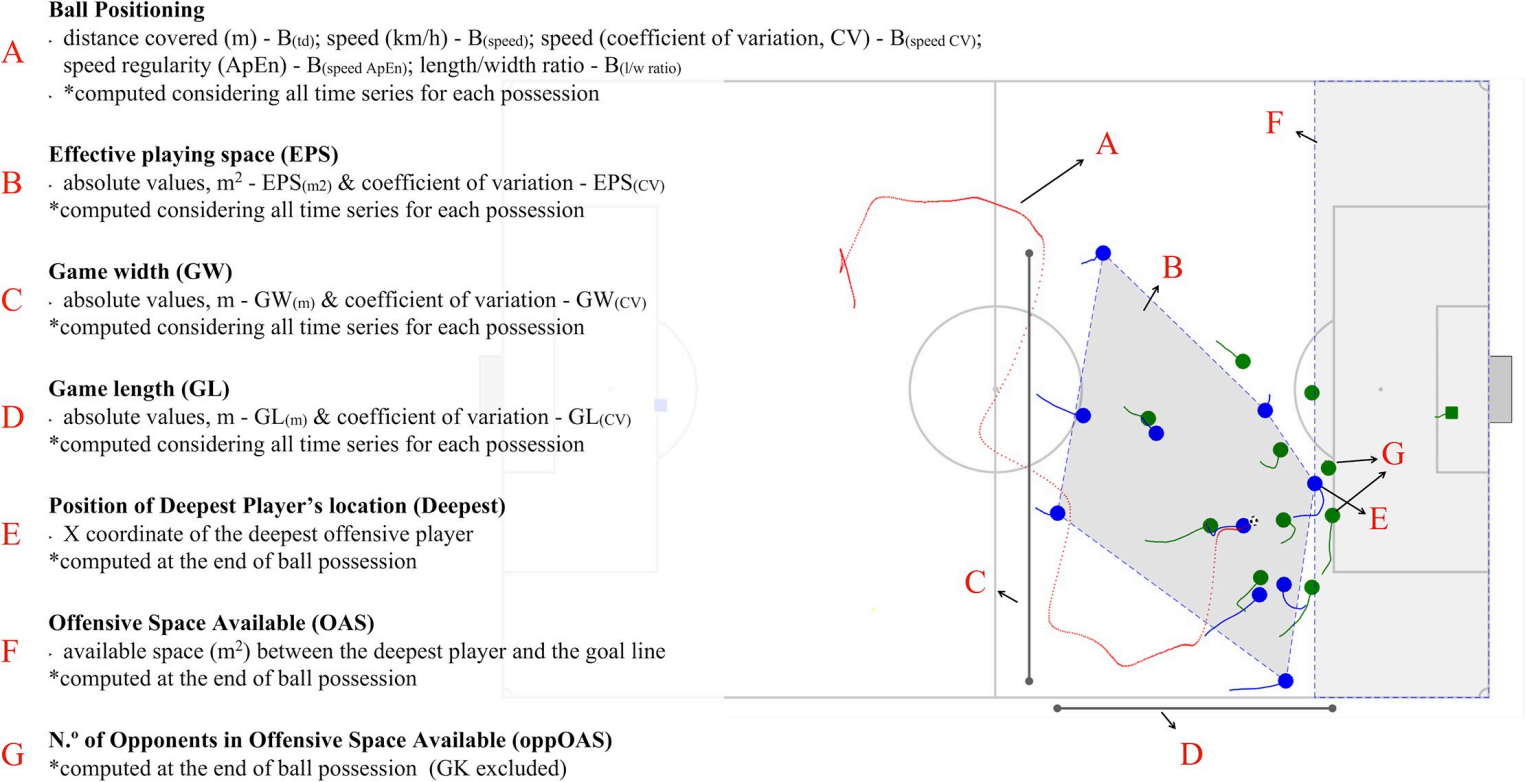
Positional tactical variables represented in a real match frame animation.

The considered number of ball possessions was selected, processed and analysed based on the following inclusion criteria: (i) minimum possession duration of 8 s (for nonlinear computations purposes described in the next paragraph); (ii) all possessions without set pieces. The ball possessions for each team started when a player performed an action with the ball (following an action from an opponent’s player or a game interruption) and ended when the ball was lost or when the ball went out of the pitch after a shot. For each ball possession sequence, several variables were calculated based on: ball position; team space in possession; game space (comprising the outfield players of both teams); position and space at the end of ball possession. Fig 1 depicts all processed variables, abbreviations, and the corresponding illustrations in a real match frame animation. Complementary, the attached supplement video shows an animation produced with underlying dynamics of each variable.

The absolute mean values and the coefficient of variation (CV%) were calculated for each variable. The normalized approximate entropy (ApEn), a measure of regularity derived from the original ApEn [27], was calculated for the speed of the ball in each possession sequences. These methods allow the comparison between ball possessions sequences with time series with different lengths. ApEn technique was used to assess regularity or predictability of the ball speed time series where input values for computations were 2.0 to the vector length (m) and 0.2 standard deviations to the tolerance factor (r) [40]. The outcome range between 0 and 2 (arbitrary units) and lower values represented more repeatable, regular, predictable and less chaotic sequences of data points [28, 41]. Also, and according to previous calculation recommendations [40], to increase the reliability of normalized ApEn computation, only ball possessions larger than eight seconds (8 seconds × 25 Hz = 200 frames) were considered for further analysis. From a processing approach, ApEn expresses the probability that the configuration of one segment of data in a time series will allow the prediction of the configuration of another segment of the time series a certain distance apart [42]. It permits to identify if the ball displacement trajectories express a regular and predictable pattern which may, in turn, provide information about dynamical behaviors [23, 43].

### Data analysis

The variables extracted from each ball possession sequence were analysed according to the quality of the opponent (TOP or BOTTOM). The descriptive outcomes were graphically represented using split-violin plots and absolute values were presented in tables. Statistical comparisons were carried with both magnitude-based decisions and null-hypothesis analysis. For the MBI, and prior to the comparisons, all processed variables were log-transformed to reduce the non-uniformity of error. Within, non-clinical inferences were assessed via differences in group means, expressed in percentage with 95% confidence limits (CL). The threshold for a change to be considered practically important (the smallest worthwhile difference) was 0.2 times the standardisation estimated from between-subject standard deviation. The following magnitudes of clear effects were considered: <0.5%, most unlikely; 0.5–5%, very unlikely; 5–25%, unlikely; 25 to 75%, possibly; 75% to 95% likely; 95% to 99%, very likely; and >99% most likely large [44, 45]. Also, the comparisons were assessed via standardized mean differences and respective 95% confidence intervals. Thresholds for effect sizes statistics were 0.2, trivial; 0.6, small; 1.2, moderate; 2.0, large; and >2.0, very large [46]. For null-hypothesis analysis, and after the assumption of normality and homogeneity of the data, an independent t-test was conducted to evaluate the differences in variables for each comparison context and statistical significance was set at *p* < .05.

A factor analysis was performed to define the underlying structure among variables according to the considered contexts (playing against TOP and BOTTOM opponents). The principal components analysis method (PCA), with the Varimax rotation, was preferred since it is the most appropriate when data reduction is paramount. The Bartlett’s test of sphericity was computed to provide the statistical significance that the correlation matrix has significant correlations among at least some of the variables. The measure of sampling adequacy was also developed with Kaiser-Meyer-Olkin (KMO) and computed to evaluate the appropriateness of applying factor analysis, considering that values above .50 for the entire matrix or an individual variable indicate appropriateness. The number of factors to be retained was based on eigenvalues (greater than 1.0) and that explained higher than 60% of percentage of variance. Afterward, although factor loadings of ±.30 to ±.40 are minimally acceptable, values greater than ±.60 were considered for practical significance (for all processing decisions please see Hair and colleagues [47]. MBI were carried using a specific spreadsheet to compare two group means [48]. The independent t-test and the PCA computations were conducted using the Statistical Package for the Social Sciences software (IBM Corp. Released 2016. IBM SPSS Statistics for Macintosh, Version 24.0. Armonk, NY: IBM Corp). The graphical representations were processed using R [49] with open-source R-packages for performing the split-violins [50] and the 3D rotated plots [51].

## Results

**Fig 2:**
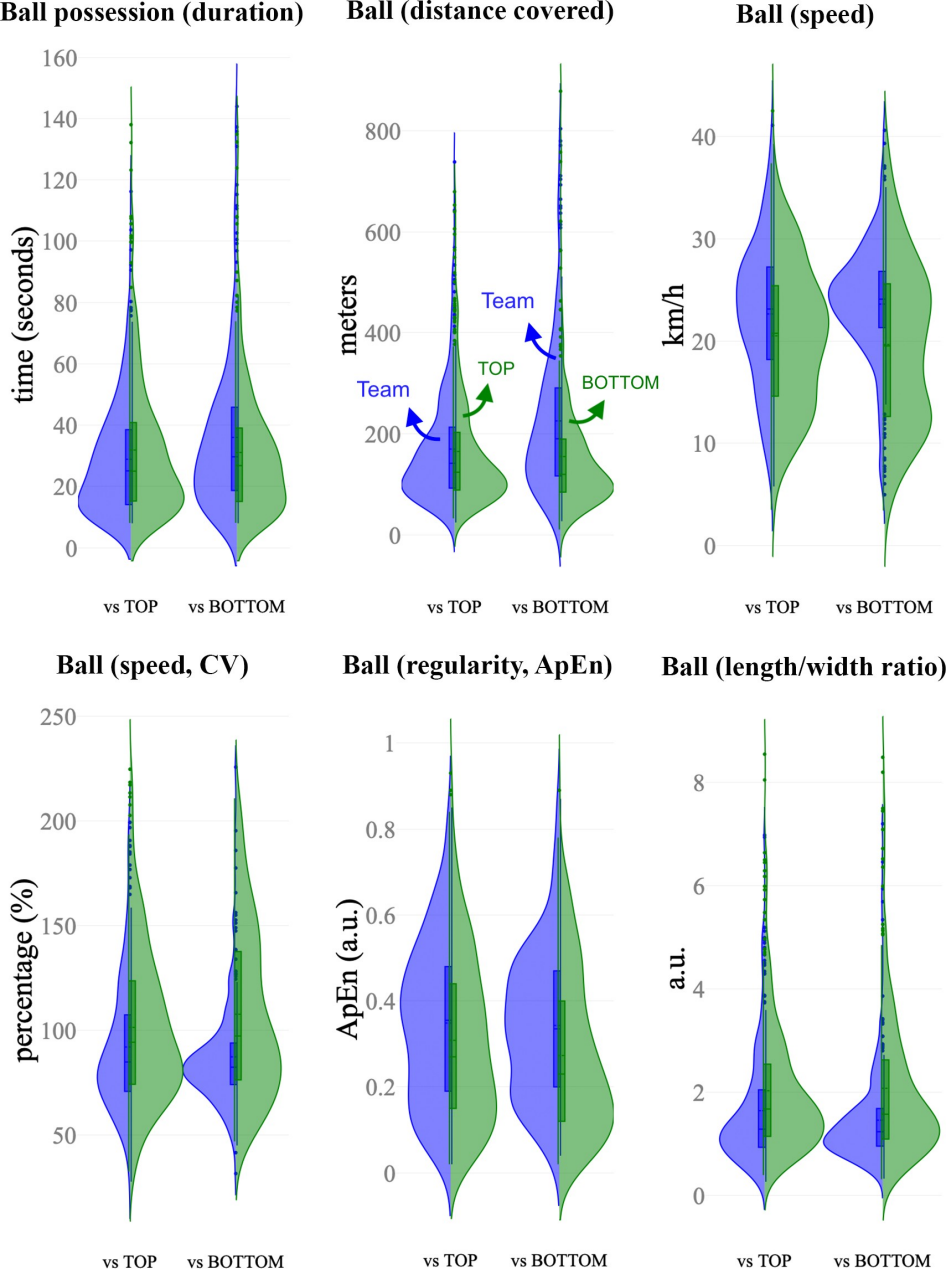
Split-violin plots showing the distribution of the computed variables based on ball position. These split-violin plots combine box plot indicating first and third quartiles and vertical lines 10th and 90th percentiles. Blue colour represents the data sets of the team while green is assigned to the opponents.

**Fig 3:**
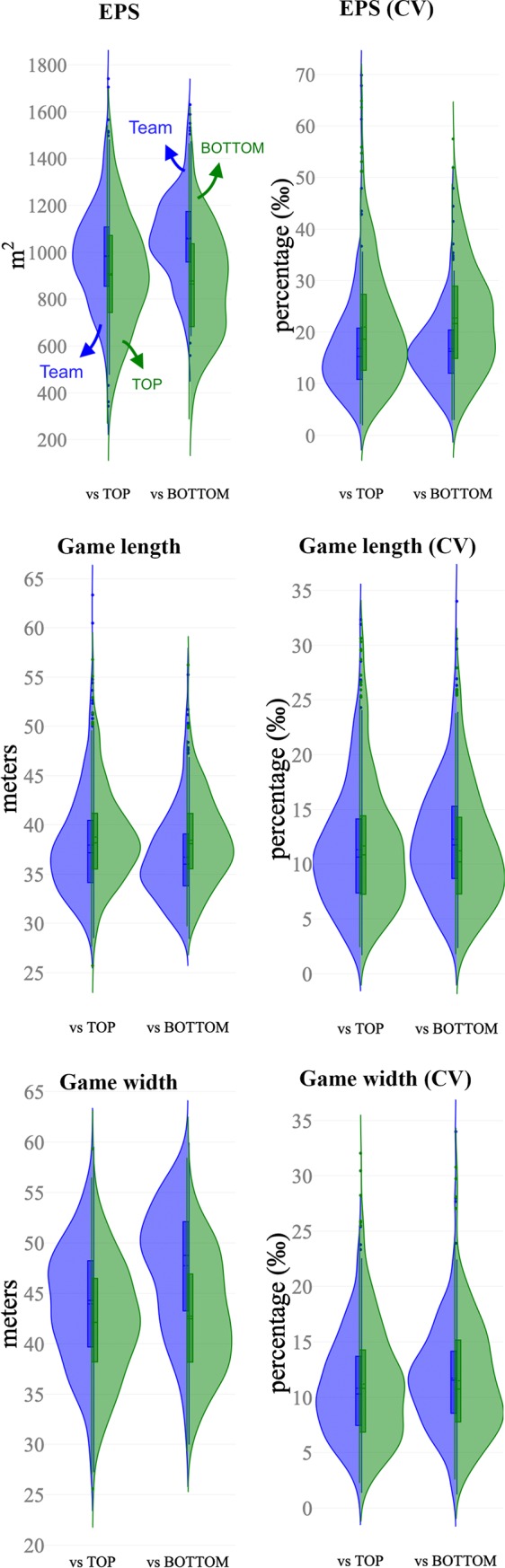
Split-violin plots showing the distribution of the computed variables based on teams’ space occupation. These split-violin plots combine box plot indicating first and third quartiles and vertical lines 10th and 90th percentiles. Blue colour represents the data sets of the team while green is assigned to the opponents.

**Fig 4:**
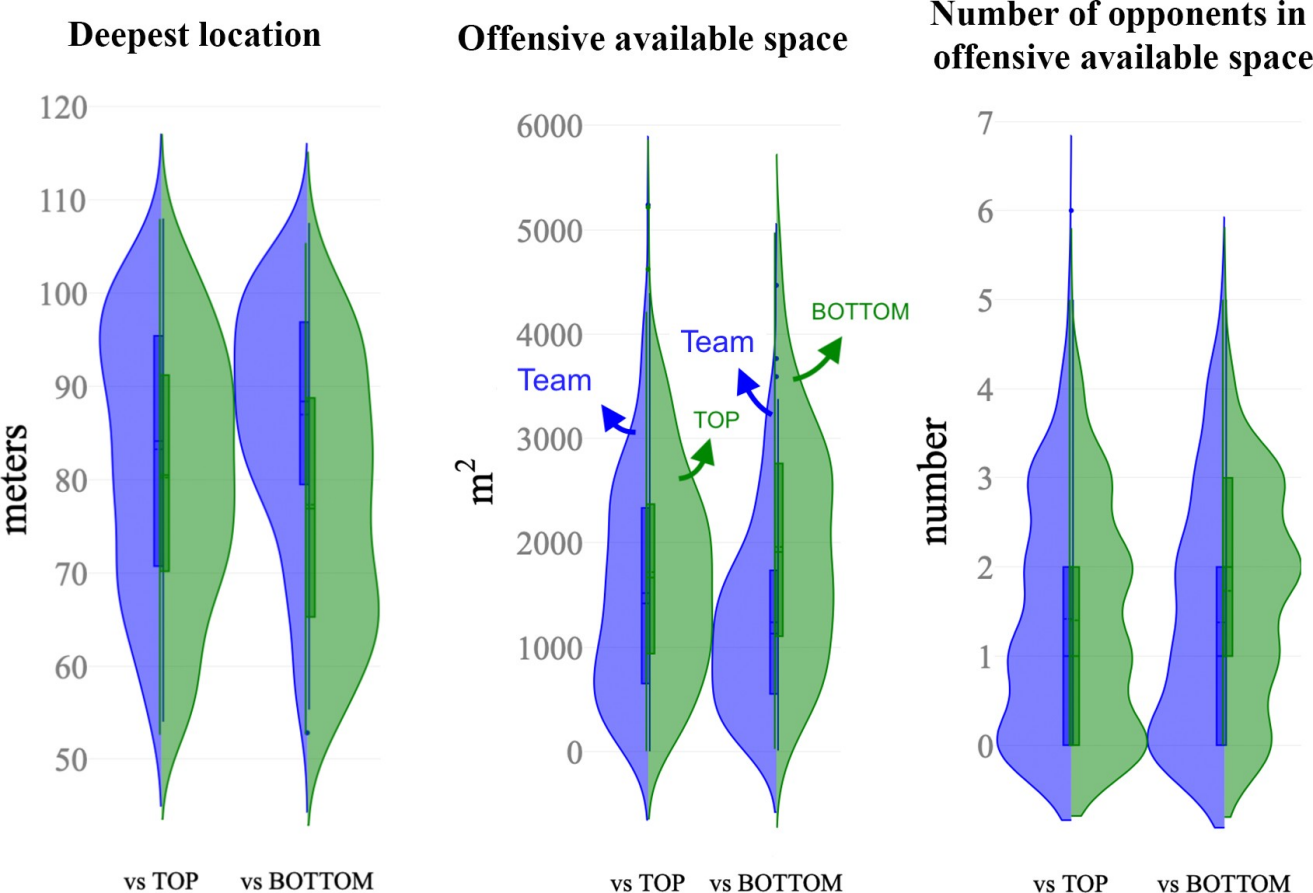
Split-violin plots showing the distribution of the computed variables based on position and space at the end of ball possession. These split-violin plots combine box plot indicating first and third quartiles and vertical lines 10th and 90th percentiles. Blue colour represents the data sets of the team while green is assigned to the opponents.

**Fig 5:**
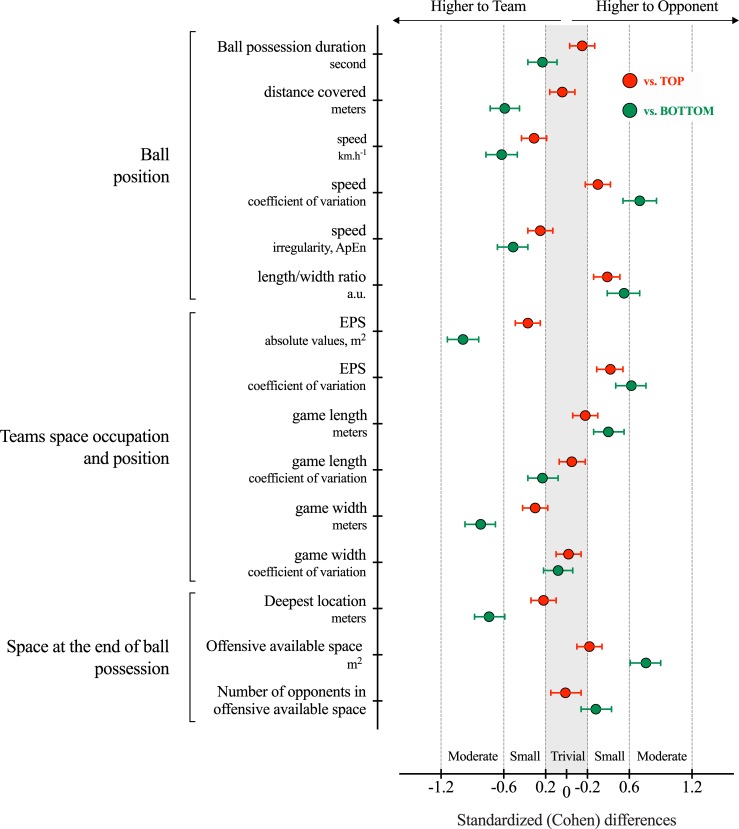
Standardized (Cohen’s d) differences computed variables according to the quality of the opponents. Error bars indicate uncertainty in true mean changes with 95% confidence intervals.

**Fig 6:**
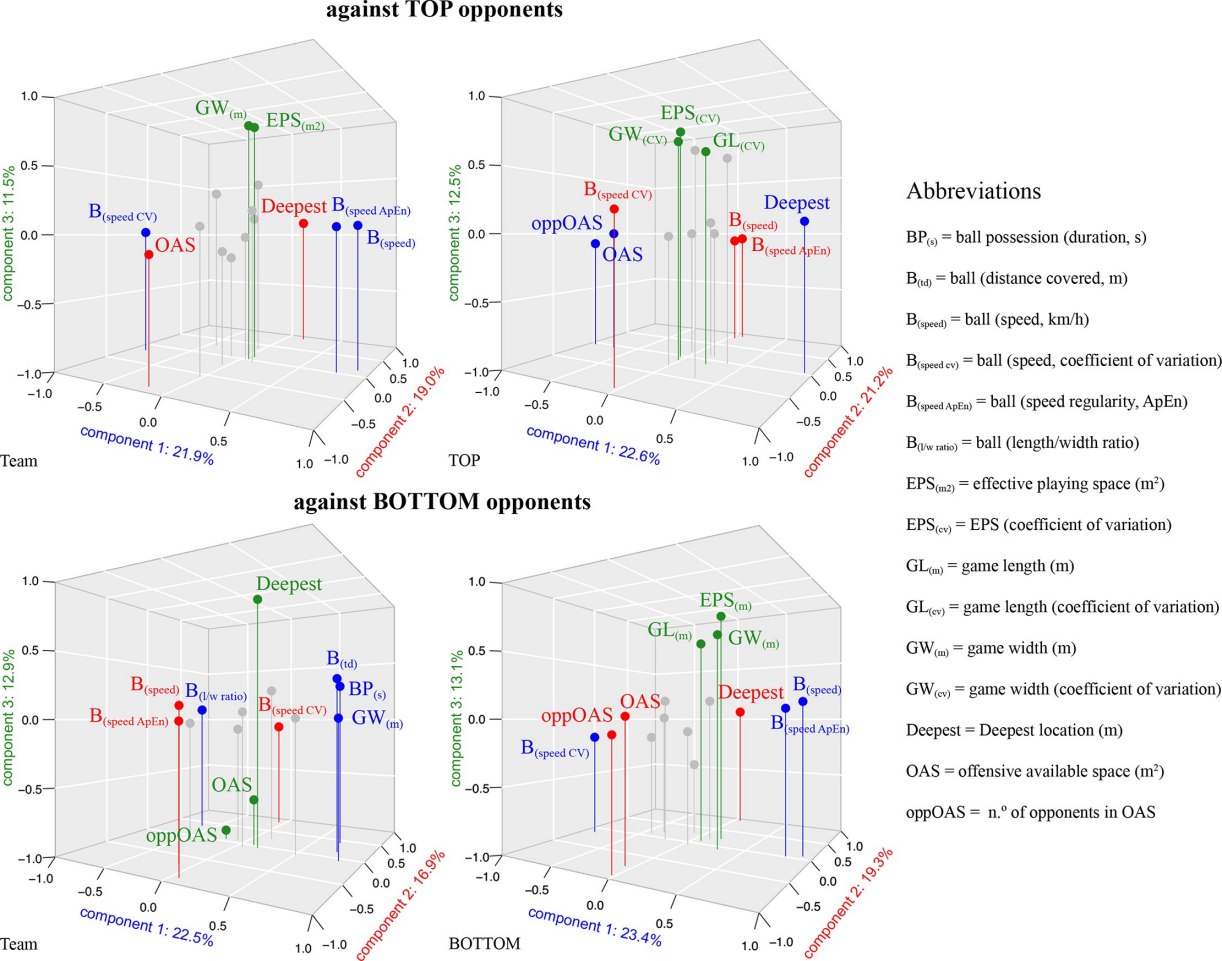
Component factors of the factor analysis (principal component methods) in 3D space according to the quality of the opponents. Variables in blue are related with 1st component, red with 2nd component and green with the 3rd component.

Figs 2, 3 and 4 depict the distribution of the computed variables based on ball position, teams space occupation and position, and space at the end of ball possession, respectively. Table 1 and Table 2 show the descriptive and inferential analysis between the matches played against BOTTOM and TOP opponents, respectively. As a complement, Fig 5 presents the standardized (Cohen’s d) differences. Table 3 and Table 4 present the component factor loadings, component statistics, Bartlett’s test of sphericity and Kaiser-Meyer-Olkin (KMO) measure of sampling adequacy of the factor analysis (principal component methods) for considered variables in the games against TOP and BOTTOM opponents, respectively. Finally, Fig 6 shows the component factors of the factor analysis in 3D space according to the quality of the opponents.

**Table 1:** Descriptive (mean±SD) and inferential analysis for the considered variables in the games against TOP opponents.

Variables	Team (n = 373)	TOP opponent (n = 369)	mean differences (%); ±95CL	Practical inferences	*t-test*	*p*
Ball possession (duration, s)	28.87±18.36	31.88±22.52	7.4; ±8.3	likely trivial	2.00	**.046**
Ball (distance covered, m)	169.69±108.8	164.84±114.71	-4.2; ±6.9	very likely trivial	-0.59	.555
Ball (speed, km/h)	22.65±6.82	20.51±7.05	-10.8; ±4.0	likely -	-4.21	**< .001**
Ball (speed, coefficient of variation %)	92.13±31.11	102.46±38.65	9.7; ±4.6	likely +	4.02	**< .001**
Ball (speed irregularity, ApEn)	0.35±0.18	0.31±0.19	-17.0; ±7.5	likely -	-2.89	**.004**
Ball (length/width ratio)	1.67±1.20	2.08±1.45	25.9; ±9.1	very likely +	4.19	**< .001**
EPS (m^2^)	984.95±212.22	908.8±242.02	-9.0; ±2.9	very likely -	-4.56	**< .001**
EPS (coefficient of variation %)	16.82±9.01	20.99±11.17	22.4; ±8.2	very likely +	5.60	**< .001**
Game length (m)	37.94±5.25	38.74±4.79	2.3; ±1.6	possibly +	2.18	**.030**
Game length (coefficient of variation %)	11.31±5.44	11.66±5.86	2.9; ±6.5	very likely trivial	0.84	.403
Game width (m)	43.94±5.89	42.13±5.87	-4.2; ±1.6	likely -	-4.20	**< .001**
Game width (coefficient of variation %)	10.81±4.45	11.19±5.31	1.0; ±5.9	very likely trivial	1.04	.299
Deepest location (m)	83.24±13.95	80.22±13.92	-3.7; ±2.1	possibly -	-2.96	**.003**
Offensive available space (m^2^)	1517.78±1013.78	1718.93±996.81	23.5; ±14.5	possibly +	2.73	**.007**
N.º of opponents in offensive available space	1.42±1.30	1.40±1.22	-0.3; ±7.3	very likely trivial	-0.17	.863

Note. Values in bold represent significant differences at *p* < .05. Abbreviations: CL = confidence limits; EPS = effective playing space;— = decrease; + = increase.

**Table 2:** Descriptive (mean±SD) and inferential analysis for the considered variables in the games against BOTTOM opponents.

Variables	Team (n = 343)	BOTTOM opponent (n = 328)	mean differences (%); ±95CL	Practical inferences	*t-test*	*p*
Ball possession (duration, s)	36.79±28.34	31.09±21.57	-13.9; ±8.0	possibly -	-2.61	**0.009**
Ball (distance covered, m)	227.45±154.2	154.74±117.93	-31.7; ±6.3	most likely -	-6.11	**< .001**
Ball (speed, km/h)	23.57±5.86	19.67±7.85	-20.7; ±4.4	most likely -	-6.54	**< .001**
Ball (speed, coefficient of variation %)	87.67±25.04	110.16±43.98	20.8; ±5.6	most likely +	7.32	**< .001**
Ball (speed regularity, ApEn)	0.34±0.17	0.27±0.19	-29.6; ±7.1	most likely -	-4.52	**< .001**
Ball (length/width ratio)	1.46±0.90	2.07±1.48	32.0; ±10.7	most likely +	5.85	**< .001**
EPS (m^2^)	1058.62±188.27	864.21±222.94	-19.9; ±2.7	most likely -	-10.93	**< .001**
EPS (coefficient of variation %)	17.03±8.30	22.74±10.37	31.2; ±9.1	most likely +	7.05	**< .001**
Game length (m)	36.70±4.52	38.42±4.36	4.8; ±1.7	very likely +	4.48	**< .001**
Game length (coefficient of variation %)	12.28±5.28	11.22±5.28	-10.3; ±6.2	possibly -	-2.31	**.022**
Game width (m)	47.75±6.04	42.74±5.8	-10.6; ±1.7	most likely -	-9.75	**< .001**
Game width (coefficient of variation %)	11.90±5.52	11.49±5.06	-3.7; ±6.5	likely trivial	-0.90	.371
Deepest location (m)	86.97±12.34	77.3±13.97	-11.6; ±2.1	most likely -	-8.50	**< .001**
Offensive available space (m^2^)	1238.24±856.02	1960.55±1074.61	79.5; ±23.6	most likely +	8.63	**< .001**
N.º of opponents in offensive available space	1.38±1.34	1.73±1.16	4.8; ±3.4	likely +	3.26	**0.001**

Note. Values in bold represent significant differences at *p* < .05. Abbreviations: CL = confidence limits; EPS = effective playing space;— = lower; + = higher.

**Table 3:** Component factor loadings, component statistics, Bartlett’s test of sphericity and Kaiser-Meyer-Olkin measure of sampling adequacy of the factor analysis (principal component methods) for considered variables in the games against TOP opponents.

Variables	Component factors (Team)					Component factors (TOP)				
	1	2	3	4	5	1	2	3	4	5
Ball possession (duration, s)	-.427	.282	.367	.556	-.373	.315	-.443	.580	.188	-.429
Ball (distance covered, m)	-.054	.371	.437	.569	-.366	.359	-.018	.580	.313	-.453
Ball (speed, km/h)	.900*	.148	.071	.005	.146	.087	.904*	-.055	.137	.103
Ball (speed, coefficient of variation %)	-.871*	-.170	.021	.078	-.038	-.043	-.887*	.161	-.098	.046
Ball (speed regularity, ApEn)	.783*	.021	.056	-.037	-.204	.049	.807*	-.069	.080	-.108
Ball (length/width ratio)	-.123	.029	-.186	-.061	.745*	-.008	-.277	-.026	-.309	.590
EPS (m^2^)	.057	.097	.877*	-.052	.323	.177	.118	.002	.896*	.192
EPS (coefficient of variation %)	-.053	-.221	-.127	.783*	.184	-.063	-.019	.829*	-.118	.061
Game length (m)	.112	-.028	.191	.005	.826*	.138	.129	.085	.317	.785*
Game length (coefficient of variation %)	-.108	.406	.135	.413	.266	.216	-.113	.626*	-.199	.250
Game width (m)	.053	.016	.880*	.031	-.236	-.047	.147	.002	.861*	-.174
Game width (coefficient of variation %)	.030	.005	-.019	.771*	-.158	-.023	-.122	.735*	.171	-.093
Deepest location (m)	.165	.903*	.107	.034	-.042	.938*	.049	.090	.020	-.029
Offensive available space (m^2^)	-.188	-.904*	-.134	-.022	.045	-.940*	-.047	-.095	-.040	.016
N.º of opponents in offensive available space	-.023	-.559	.057	.059	-.013	-.723*	-.056	.004	-.055	-.092
**Eigenvalues**										
Total	3.290	2.854	1.721	1.520	1.447	3.386	3.178	1.873	1.361	1.326
% of variance	21.9	19.0	11.5	10.1	9.6	22.6	21.2	12.5	9.1	8.8
Cumulative %	21.9	41.0	52.4	62.6	72.2	22.6	43.8	56.2	65.3	74.2
**Bartlett’s test of sphericity**										
χ^2^	3952.0					3797.0				
*p*	< .001					< .001				
**Kaiser-Meyer-Olkin measure of sampling adequacy**	.57					.63				

Note: (*) Bold represent loadings greater than ±.60.

**Table 4:** Component factor loadings, component statistics, Bartlett’s test of sphericity and Kaiser-Meyer-Olkin measure of sampling adequacy of the factor analysis (principal component methods) for considered variables in the games against BOTTOM opponents.

Variables	Component factors (Team)					Component factors (BOTTOM)				
	1	2	3	4	5	1	2	3	4	5
Ball possession (duration, s)	.687*	.418	.273	.323	-.042	-.350	.180	.014	.801*	.293
Ball (distance covered, m)	.752*	.164	.312	.323	-.020	.111	.166	.154	.823*	.273
Ball (speed, km/h)	.060	-.902*	.085	.083	.139	.915*	.084	.132	-.032	-.026
Ball (speed, coefficient of variation %)	-.072	.863*	-.065	-.014	-.108	-.893*	-.120	-.159	.011	.019
Ball (speed regularity, ApEn)	-.119	-.671*	-.014	-.146	-.177	.814*	.017	.081	-.014	-.114
Ball (length/width ratio)	-.642*	.360	.086	.006	.216	-.425	.099	-.163	-.455	.151
EPS (m^2^)	.515	-.082	.013	-.281	.659*	.185	.222	.859*	.174	.027
EPS (coefficient of variation %)	-.079	.079	-.082	.785*	.107	.035	-.055	-.099	.023	.810*
Game length (m)	-.410	-.126	-.028	.008	.797*	.068	.086	.617	-.551	.123
Game length (coefficient of variation %)	.154	.250	.241	.362	.433	-.097	.276	-.400	.142	.443
Game width (m)	.834*	-.048	.012	-.344	.107	.297	-.068	.651*	.403	-.153
Game width (coefficient of variation %)	.049	-.072	.062	.753*	-.153	-.207	-.023	.149	.178	.743*
Deepest location (m)	.168	-.044	.937*	.063	-.070	.051	.938*	.070	.072	-.024
Offensive available space (m^2^)	-.189	.066	-.928*	-.069	.073	-.034	-.932*	-.102	-.104	-.027
N.° of opponents in offensive available space	.108	.011	-.639*	.088	-.198	-.069	-.736*	.021	.009	-.004
**Eigenvalues**										
Total	3.379	2.536	1.939	1.578	1.361	3.512	2.890	1.963	1.329	1.215
% of variance	22.5	16.9	12.9	10.5	9.1	23.4	19.3	13.1	8.9	8.1
Cumulative %	22.5	39.4	52.4	62.9	71.9	23.4	42.7	55.8	64.6	72.7
**Bartlett’s test of sphericity**										
χ^2^	3170.5					2414.2				
*p*	< .001					< .001				
**Kaiser-Meyer-Olkin measure of sampling adequacy**	.56					.62				

Note: (*) Bold represent loadings greater than ±.60.

The changes in position-derived variables showed different trends according to the quality of the opponents. When facing TOP opponents, trivial to small differences were observed in all the considered variables. The ball speed and ApEn were likely lower (p < .01, small effect) in TOP opponents while higher values were observed to CV% and length/width ratio (p < .001, small effect). The team space occupation and position show likely small higher values in EPS (p < .001) when the team was in possession (Team = 984.95±212.22 m^2^ vs TOP = 908.8±242.02 m^2^) with lower CV% (p < .001, small effect). The team’s possessions tended to end closer to the opponents’ goal (nearly trivial effect, p = .003, Team = 83.2±13.0 m vs. TOP opponents = 80.2±13.9 m).

When analysing matches against BOTTOM opponents, the variables changed substantially (differences from small to nearly large according to Cohen’s d effects). The ball possessions of the team were higher in duration (higher 13.9%; ±8.0%, p = .009, small effect) and higher in both distance covered and ball speed (p < .001, moderate effect) compared to the ball possessions of the BOTTOM teams. This trend was followed by moderate lower ball speed’s variability (lower 20.8%; ±5.6%, p < .001) and a moderate higher ball speed’s ApEn during the possession (29.6%; ±7.1%, p < .001). The variables related to team space occupation and position presented moderate higher values in the effective playing space of the team (Team = 1058.6±188.3 m^2^ vs BOTTOM = 862.9±223.6 m^2^) with a moderate lower CV% (lower 31.2%; ±9.1%, p < .001). When the team was in possession, the game length was lower than their opponents (Team = 36.70±4.52 m, BOTTOM = 38.42±4.36 m, p < .001, small effect), however, the game width was 10.6%; ±1.7% higher (Team = 47.75±6.04 m, BOTTOM = 42.74±5.8 m, p < .001, moderate effect). The possessions tended to end closer to the opponents’ goal when team played against BOTTOM opponents (Team = 86.9±12.3 m vs. BOTTOM = 77.2±13.9 m) than against TOP opponents (nearly trivial effect, Team = 83.2±13.0 m vs. TOP opponents = 80.2±13.9 m, p < .001, moderate effect). The number of opponents in the offensive available space was lower than BOTTOM opponents (i.e. higher number of defenders in the offensive area).

The principal components model accounted for 72.2% of the total variance for the team and 74.2% to the TOP opponents, with five component factors extracted for each. For the team, the first component (21.9%) consisted of ball speed (loading = .900), ball speed CV% (-.871) and ball speed ApEn (.783); while the second component (19.0%) was related to the deepest location (.903) and the offensive available space (.904); and the third component (11.5%) associated EPS (.877) and game width (.880). For TOP teams the first component (22.6%) consisted of the deepest location (.938), offensive available space (-.938) and the number of opponents in offensive available space (-.723); the second component (21.2%) associated ball speed (.904), ball speed CV% (-.887) and ball speed ApEn (.807); and the third component highlighted the relation of the EPS coefficient (.829), game length (.626), and game width (.735) (see Table 3 and Fig 6, upper panel, for complementary information).

In the analysis of the team against BOTTOM opponents, the principal component model accounted for 71.9% and 72.7% of the total variance, respectively. For the team, the first component (22.5%) consisted of the duration of possession (.687), the distance covered by the ball (.752), length/width ratio of the ball displacement (-.642) and game width (.834); the second component (16.9%) associated ball speed (-.902), ball speed CV% (.863), and ball speed ApEn (-.671); and the third component (12.9%) consisted of the deepest location (.937), offensive available space (-.928) and the number of opponents in offensive available space (-.639). For BOTTOM teams, the first component (23.4%) consisted of ball speed (.915), ball speed CV% (-.893) and ball speed ApEn (.814); the second component (19.3) highlighted the association of the deepest location (.938), offensive available space (-.932), and the number of opponents in offensive available space (-.736); and the third component (13.1) related the EPS (.859), and game width (.651) (see Table 4 and Fig 6, lower panel, for complementary information).

## Discussion

The aim of this study was twofold: (i) to describe the collective behavioural patterns that relies upon players’ positioning in interaction with teammates, opponents and ball position; (ii) and to define the underlying structure of the derived variables using factorial analysis. As was hypothesized, the quality of opponents promoted substantial variations in team tactical behaviour. The descriptive analysis may provide key information to plan, design and execute representative tasks during the training process. In addition, the PCA revealed the determinants of collective tactical behaviour that coaches should consider when designing representative training tasks.

Improving performance requires that coaches use a multifactorial training process that integrates information from physical, technical and tactical game requirements [12, 52]. The alignment on teammates’ performance with the collective strategic plan as well considering the specificities of opponent team is fundamental to ensure the transfer between practice and competition [34–36]. Consequently, the daily challenge for coaches’ intervention is to identify, manipulate and monitor the variations in spatial-temporal information constraints within the simulated scenarios [36]. Despite some very interesting approaches that highlight the importance of representativeness in youth players’ training [53, 54], it is still not clear how to gather the information on the individual and collective responses and the different levels of contextual unpredictability that the training process should embrace to reflect the competitive reality. In this sense, a deeper analysis of match outcomes might help to guide coaches in the planning, designing and execution of more ecological training tasks. In other words, the practice should promote the emergence of match specific behaviours and favour a high degree of transferability of players’ behaviour from the training drills to competition [34].

Previous research explored the effect of teams’ performance level in spatial variables revealing that there is a higher use of the width of the pitch in detriment to the length, and that first division teams display greater depth than second division teams [55]. Also, results suggested that the playing space is influenced by the ball location. In fact, playing lengths tend to decrease while widths tend to increase as the ball moves from the goal area to the midfield zones [56]. Accordingly, the rectangle comprising all the outfield players have been characterized by lower values of length and higher values of width when the ball is in the central area of the pitch [57]. While these studies have added important information regarding space occupation, also, important practical information may be picked to design playing area dimensions, if it is considered the dynamics between the opposing teams when accounting to the quality of opposition. In the present study, different playing spaces emerged when comparing the players performance according to the opposition quality. For example, when facing TOP opponents, the possessions used ~38 meters game length per ~43 meters game width with 12% coefficient of variation (%). This information can be directly applied in the design of training tasks. When the scope of the task is working on functional solutions near to the scoring area, the definition of playing space constraints can be parametrized by these results. Additionally, the possessions last ~28 seconds and at the end of the ball possession, the deepest location of the offensive player was at ~83m of the pitch length. These behavioural features reflect the temporal adaptations to new spatial structures and configurations generated by players’ sub-groups interactions as match situations dynamically change [22, 23, 58, 59]. These results can be used as spatiotemporal guidelines with higher transferability from match to training tasks because of the high level of representativeness. Overall, the final aim is to potentiate the development of players’ tactical awareness through the creation of training tasks based in contexts that stimulates decision-making under strong time-pressure.

**Fig 7:**
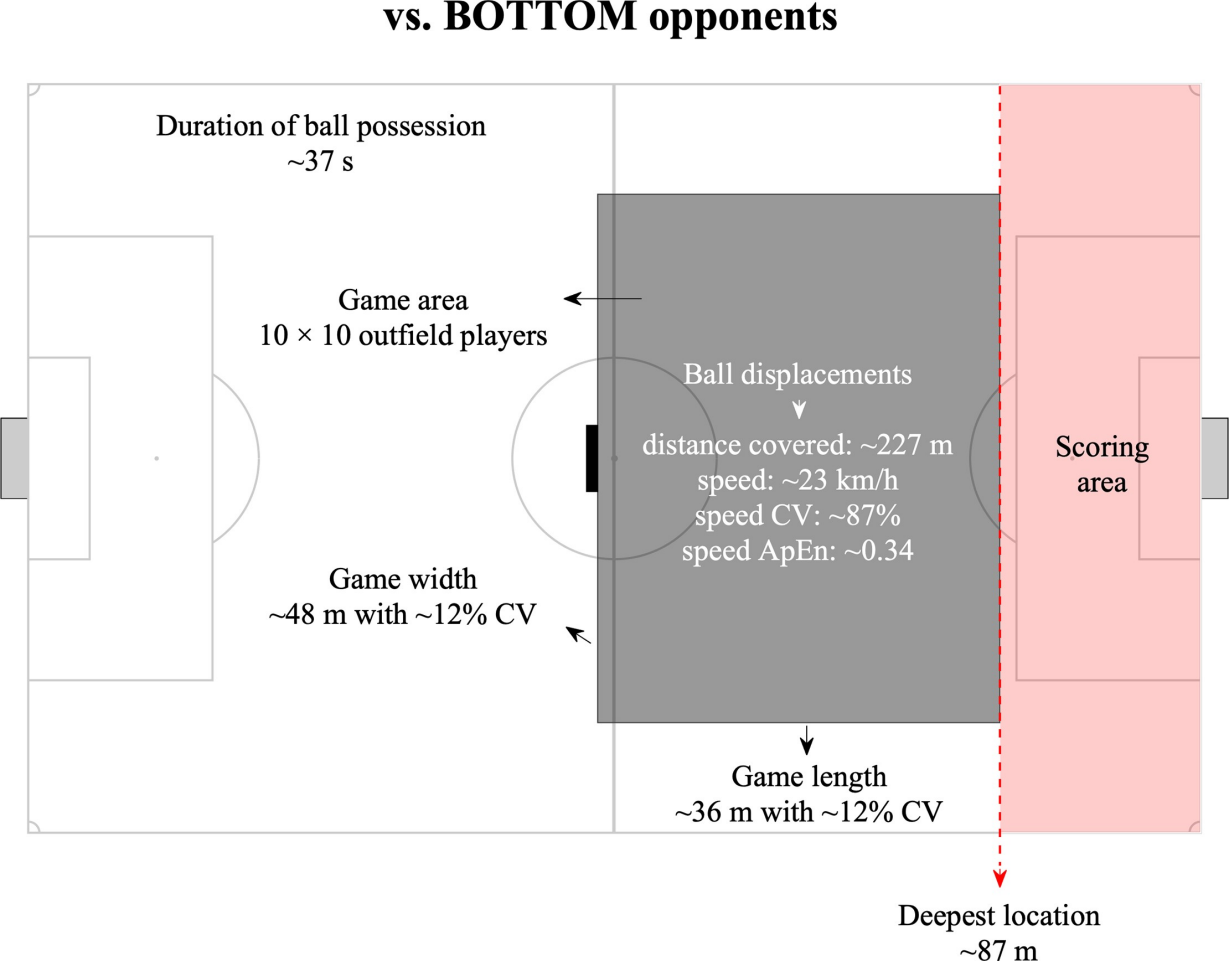
A practical application example of the results when the team faced BOTTOM opponents. All the spatial references are real values. The dimension of the pitch is 105 × 68 meters.

The length, width and surface area have been used before by exploring the effects of several manipulations on the players' performance during small-sided games [60–62]. The same metrics have been also analysed in match context according to the quality of the opposition, and the results showed higher values for offensive situations when playing against weak teams [63]. In this study, we updated this information with complementary insights. Against BOTTOM opponents, the deepest location increases, consequently making the scoring area smaller to the team. The playing game space increased in width and decreased in length. In addition, the duration of ball possession increased considerably (~37 seconds) and the ball speed ApEn was higher, suggesting lower levels of regularity. The combination of these match features is associated with lower ratio of the length/width ball displacement, suggesting passing actions towards the lateral direction. This might be the result of defensive compactness, that requires the use of the pitch width to create space. In fact, previous research has shown that teams of lower quality are likely to defend closer to their target to restraint space and time [63], which may led to higher use of the pitch width by the offensive team to break the defensive stability and create space for shooting opportunities. Practical applications of these results can be observed in Fig 7. The spatial references (game area, scoring area, etc.) represent real values and may be used as baseline information to create task structural boundaries. Accordingly, coaches may use complementary constraints (e.g. number of touches allowed to increase/decrease the ball speed, floater players to give higher depth or width to the possession, the goal of the exercise to manipulate the ball possession duration, etc.) to develop and practice team strategies and boost task representatives.

Previous research has shown that weaker teams are likely to adopt a more direct playing style as pattern of play [64]. While this behaviour has been attributed to their lower ability to maintain the ball possession, in turn, the results from this study may provide additional information. Accordingly, from the perspective of the BOTTOM teams in possession, players face a higher pressure since their EPS is small, even offensively (864.21±222.94 m^2^). This pressure affected the deepest location that considerably decreased (~77 meters), creating an open space in the defensive back (of the TOP opponents). This open space may afford the BOTTOM teams to use a more direct playing style to explore. In fact, the increased ball length per width ratio (~2 arbitrary units) seems to support this idea. Nevertheless, is important to highlight some spatiotemporal boundary condition so that players’ can adequately reproduce during the training the interaction dynamics that are mediated by match environmental information.

On integrating association mining with PCA, this study extends the application and understanding of match analysis by compression statistical mechanism. Previous studies have already explored this method which frequent patterns are analyzed for their interrelationship in order to generate association in players’ performances [65–69]. Results related to notational analysis suggested shots, shots on goal, playing time with ball possession and percentage of ball possession as the most important variables to discriminate winning teams from drawing and losing [68]. Additionally, winning teams have already been reported to exhibit different and consistent profiles from drawing and losing teams, mainly discriminated by their ability to recover the ball in zone 2 (close to middle line in the defensive half), and to organize the offense using penetrative passes to the penalty area to increase the number of shots and, consequently, goals [66]. The present study extends the PCA approach to the spatiotemporal variables, aiming to define its underlying structure in terms of emergent collective behaviour, as well as to model how the quality of opposition affects these relations. The outcome can be used as key information to understand how the manipulation of tasks will change the variables’ association. Also, it may be used by coaches to verify whether the essence of tactics or playing style practiced in sessions is performed during competition. The matches played against TOP opponents showed similar trends. The first three components explained more than 50% of the data variance, showing that the increase of ball speed and ApEn of the ball speed were associated with ball speed CV%. The increase in the deepest location at the end of ball possession generated less offensive available space, as expected, and the EPS of the team in possession was well correlated with the game space, specially the game width. Bringing this information to the training process, coaches can increase/decrease the effective playing space by changing the pitch width any consequent changes in ball movement (number of touches allowed, for example) will promote changes in ball speed variability (for magnitude, CV%, and structure, ApEn). A recent study explored the soft-assembly of tactical patterns and the timescales of positioning-derived variables that define them during a soccer match, allowing understanding the multilevel organization of tactical behaviors as defined by the timescales of evolution of collective patterns [65]. In fact, the authors showed how teams behave during a competition and how these behavioral patterns change over the course of the match, influenced by the different constraints. However, there was still a gap to be filled about the boundary conditions that could be applied to training tasks so the players’ ability to adapt to different context information would be practiced in a rate of change which reflects competition.

The PCA analysis for the matches played against BOTTOM opponents provided further relevant context information. In the team, the principal components showed a strong association among ball related variables. The ball possession duration was strongly related to the game width and distance covered by the ball, and negatively related with length/width ratio of the ball movement. Moreover, these results are connected with the descriptive analysis, where was shown that the team need to increase the width of the game in order to create open space. In fact, stronger teams with higher skilled players seem to be able to sustain the ball possession for longer time, develop longer passing sequences, thus producing more goals per possession than shorter passing sequences [64].

Further research can include all levels of performance because the current study only explored the impact of playing against top and bottom teams. Also, technical and physical performance data can be considered in a more multidimensional and integrated approach, to increase the understanding of how players base their game interactions and, thus, constitute a solid criterion for fine-tuning the training process and performance modeling.

## Conclusion

Spatiotemporal temporal patterns of play that sustain collective tactical behaviour of teams can be extracted from matches to design highly representative training tasks and provide a better understanding of the underlying processes, contributing to performance outcomes. In line with this statement, the ball-position derived variables added novel information to describe the collective behaviour patterns and should be considered into analysis. As expected, the quality of opponents promotes a great variation in team behaviour as function of possession and is presented as an important factor to be considered. When playing against TOP opponents, the team possessions lasted for ~28 seconds, in a playing space of ~38 meters of length per ~43 meters of width and with the deepest location of the offensive player at ~83m of pitch length. Against BOTTOM opponents, the deepest location increased, as well as the game width. These results seem to emerge as the team attempts to explore the pitch lateral spaces to break the defensive stability of the BOTTOM teams, leading to a decrease in the ratio of the length/width ball displacement, and an increase in the duration of ball possession (~37 seconds). From the perspective of BOTTOM teams in possession, players face a higher pressure, reflecting a small EPS. Furthermore, this open space may afford the BOTTOM team to explore a more direct playing space, which can be depicted from the increase in the ball length per width ratio (2 a.u.). The PCA provided relevant complementary information to define the underlying structure among the variables and showed a strong association with ball speed, ApEn and CV% of the ball speed. The EPS of the team in possession was correlated with the game space, especially the game width against TOP opponents. Against BOTTOM opponents, there was a strong association with ball possession duration, game width, distance covered by the ball, and length/width ratio of the ball movement. The overall approach carried in this study may serve as baseline to elaborate normative models of positioning behaviours measurements to support coaches’ operational decisions towards a holistic, representative and complex point of view that help to design highly representative training tasks.

## Supporting information

S1 Video Video animation produced from the real calculated variables for better visualization of the underlying dynamics. The video ended when the ball goes out, however, to data processing, the ball possession ended in the exact time frame when the player performed the shot (represented in Fig 1).
(MP4)
